# Experimental and Modeling High-Pressure Study of Ammonia–Methane Oxidation in a Flow Reactor

**DOI:** 10.1021/acs.energyfuels.3c03959

**Published:** 2024-01-08

**Authors:** Pedro García-Ruiz, Iris Salas, Eva Casanova, Rafael Bilbao, María U. Alzueta

**Affiliations:** Department of Chemical and Environmental Engineering, Aragón Institute of Engineering Research (I3A), University of Zaragoza, 50018 Zaragoza, Spain

## Abstract

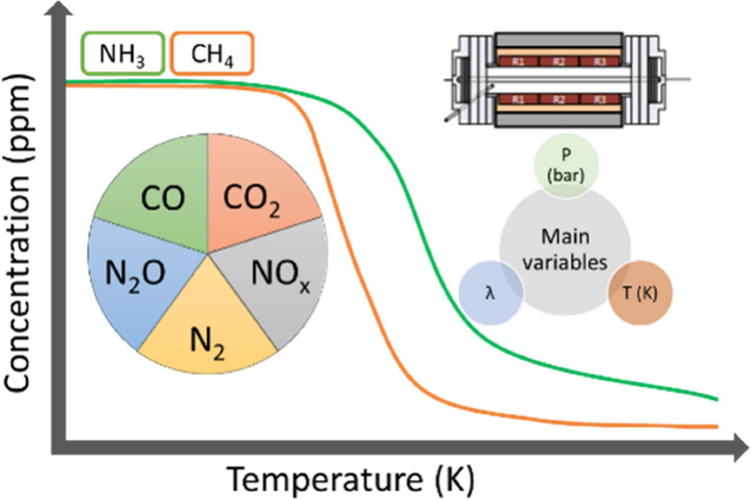

The present work deals with an experimental and modeling analysis of the oxidation of ammonia–methane mixtures at high pressure (up to 40 bar) in the 550–1250 K temperature range using a quartz tubular reactor and argon as a diluent. The impact of temperature, pressure, oxygen stoichiometry, and CH_4_/NH_3_ ratio has been analyzed on the concentrations of NH_3_, NO_2_, N_2_O, NO, N_2_, HCN, CH_4_, CO, and CO_2_ obtained as main products of the ammonia–methane mixture oxidation. The main results obtained indicate that increasing either the pressure, CH_4_/NH_3_ ratio, or stoichiometry results in a shift of NH_3_ and CH_4_ conversion to lower temperatures. The effect of pressure is particularly significant in the low range of pressures studied. The main products of ammonia oxidation are N_2_, NO, and N_2_O while NO_2_ concentrations are below the detection limit for all of the conditions considered. The N_2_O formation is favored by increasing the CH_4_/NH_3_ ratio and stoichiometry. The experimental results are simulated and interpreted in terms of an updated detailed chemical kinetic mechanism, which, in general, is able to describe well the conversion of both NH_3_ and CH_4_ under almost all of the studied conditions. Nevertheless, some discrepancies are found between the experimental results and model calculations.

## Introduction

Nowadays, the rise in the global concentration of carbon dioxide and harmful pollutants due to the growing energy demand has become a serious problem. Future sustainable scenarios involve the use of carbon-free fuels. In this sense, species such as hydrogen and ammonia play a role as possible substitutes for fossil fuels,^1^ to solve the climate change problem. An alternative solution is the use of noncarbon fuels, such as ammonia or hydrogen, as fuels or as chemical storage.^2^ Hydrogen has attracted attention as a fuel with zero CO_2_ emissions,^3^ but it has some application inconveniences associated with its high storage cost. Proof of this is the fact that storing H_2_ at high scales in practical applications implies compression to between 350–700 bar or cryogenic cooling to 20 K.^4^ For these reasons, H_2_ storage is more difficult and expensive than storing NH_3_.^5^ Moreover, following the interest in ammonia as a hydrogen carrier, there is nowadays an increased interest in ammonia as a fuel. Since ammonia is one of the most produced chemicals in the world, it exhibits a mature production technology, transportation, and storage infrastructure.^6,7^ Ammonia has been suggested as a substitute for hydrogen,^8^ and since the previous decade, initiatives to accelerate global decarbonization have increasingly focused on the use of NH_3_ as a feasible alternative fuel.^9^ Additionally, ammonia can ideally be burned in an environmentally benign way, producing N_2_ and H_2_O (R1)^10^:
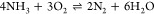
R1

At the same time, NH_3_ could potentially be a future NO_*x*_-free fuel under specific operating conditions as Shu et al.^11^ conclude, since ammonia can interact under specific conditions (fuel-lean conditions) within the combustion chamber with NO, effectively minimizing its concentration through selective noncatalytic (SNCR) reactions.^10−14^ In this way, ammonia can react with NO by the following mechanisms (NH_3_ → NO → NNH → N_2_ and NH_3_ → NO → N_2_) within the combustion chamber, as pointed out by Alzueta et al.^12^ through reactions (R2–R5):
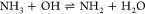
R2
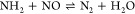
R3
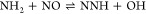
R4
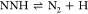
R5

However, ammonia presents some challenges for its practical implementation associated with its poor combustion characteristics, such as its high ignition temperature and low flammability,^7,10^ potential NO_*x*_ emissions,^12^ and a low laminar burning velocity which is about five times lower compared to CH_4_/air flames.^15^ Thus, the addition of CH_4_ can improve the combustion characteristics of ammonia,^14^ because it can provide a higher flame speed, and a higher thermal efficiency^16^ at the same time. The NH_3_/CH_4_ mixture also provides an advantage for CO_2_ emissions minimization compared to natural gas burning because ammonia replaces part of the hydrocarbon fuel. Under locally fuel-rich conditions within the combustion chamber, CH_4_ also can reduce the NO concentration, mainly by reburn reactions.^12^ In the NH_3_/CH_4_ mixtures, the reburn reactions occur through the generation of hydrocarbon radicals at high temperatures and fuel-rich conditions, which are able to interact with NO, through reactions (R6,R7),^17^ involving the methyl radical formed from methane (R8):
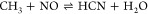
R6
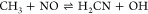
R7
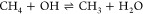
R8

The intermediate species HCN and H_2_CN formed will be converted to N_2_ or NO within the combustor chamber under specific conditions. The combined use of ammonia and methane can be beneficial for NO_*x*_ reduction through the two mechanisms described above, namely, SNCR and reburn. In the past few years, for the above reasons among others, mixtures of NH_3_ with other fuels such as CH_4_ have exhibited a strong interest to be accounted for in the transition from energy systems based on fossil fuels to others based on carbon-free fuels. A large number of experimental and modeling studies of the combustion of NH_3_/CH_4_ mixtures at low pressure (from 0.4 to 5 bar) have been reported in the literature, using different experimental set-ups such as flow reactors,^12,18−20^ different burners such as the Mckenna burner,^21^ a plate burner,^22^ a swirl burner,^8,23,24^ an axisymmetric burner,^25^ a heat flux burner,^26^ different premixed laminar burners,^27−29^ and a porous media burner,^30^ as well as combustors such as cylindrical combustor chambers,^6,16,31^ turbines such as a micro gas turbine combustor,^14^ a swirl flame combustor,^24,32−34^ and gas turbine systems,^35−38^ as well as numerical studies.^39−42^

Among the previous studies, we can find flame studies,^8,21,25^ emissions characteristics, flame propagations, flame structure, autoignition properties,^14,35,39^ laminar burning velocity and flame speed,^6,16,26,29^ and numerical studies.^6,8,11,12,14,16,18,20−25,27−30,32,34−36,39−43^

It is important to mention as well that some studies at atmospheric pressure focused on determining the effects of NO concentration on ammonia chemistry during NH_3_/CH_4_ mixtures oxidation.^12,22,27,40^ Also, the effects of CO/CO_2_ are also studied on NH_3_ oxidation in a flow reactor with the aim of addressing possible oxy-fuel combustion strategies.^18,27,44−46^

From a practical point of view, pressure is an important variable since turbines and certain engines will operate under high pressure conditions.

Increasing pressure has been reported to contribute to reduce the unburned NH_3_ and NO emission.^47^ In this sense, it is essential to examine whether the same effect happens for other reaction products, such as HCN. In this regard, studies in the literature on NH_3_/CH_4_ blends at high pressure (from 5 to 100 bar) are rather scarce, and among those we can mention a study on turbulent burning velocity and flame region in a nozzle burner at 5 bar by Ichikawa et al.,^48^ an autoignition delay time study from 20 to 70 bar and temperatures from 930 to 1140 K of different CH_4_/NH_3_ mixtures (0, 5, 10 and 50% of CH_4_ in the mixture) in a rapid compression machine by Dai et al.,^43^ an experimental and numerical study of autoignition at 1.75 and 10 bar in a shock tube, in which CH_4_/NH_3_ ratios of 0, 0.1, and 0.5 were studied,^49^ an experimental and numerical study on laminar burning velocity in a pressurized chamber up to 5 bar by Wang et al.^50^ and chemical kinetic modeling studies.^34,41^ In these studies, it was found that, at higher pressures, there is less unburned NH_3_ and lower NO emissions at the outlet of the reactor compared to what occurs at atmospheric pressure. Additionally, the ignition delay time decreases as CH_4_ is added to the mixture, even if added in a small ratio, compared to the conversion of net ammonia. The oxygen excess ratio is also important since the ignition delay time is higher at lower oxygen concentrations. Furthermore, methane has an enhancing effect on ignition due to its impact on H_2_O_2_, CH_3_O, and H_2_NO, resulting in the promoting effect of ammonia conversion under different conditions.^43,48−50^

To our knowledge, there is a lack of studies about NH_3_/CH_4_ mixture oxidation under relevant conditions carried out in plug flow reactors and high pressures (10 to 40 bar) in a variety of stoichiometries ranging from reducing to oxidizing atmospheres. In this context, the aim of this work is to extend the knowledge of NH_3_/CH_4_ mixtures oxidation using the experimental system described above, by analyzing the effect of temperature (from 550 to 1250 K), pressure (from 10 to 40 bar), oxygen excess ratio (from reducing, λ = 0.7, to oxidizing conditions, λ = 3) and CH_4_/NH_3_ ratio (0.5, 1 and 2). Experiments are performed using argon as a bath gas, which allows us to accurately determine the molecular nitrogen formed during the reaction and therefore to be able to perform nitrogen balances. Additionally, in order to understand the chemistry and product species formation, the results are interpreted in terms of a chemical kinetic mechanism for ammonia/hydrocarbon mixtures, which has been compiled from the literature and updated in the present work, and the main reaction pathways through which reactions proceed have been determined and discussed.

## Methodology

**Figure 1:**
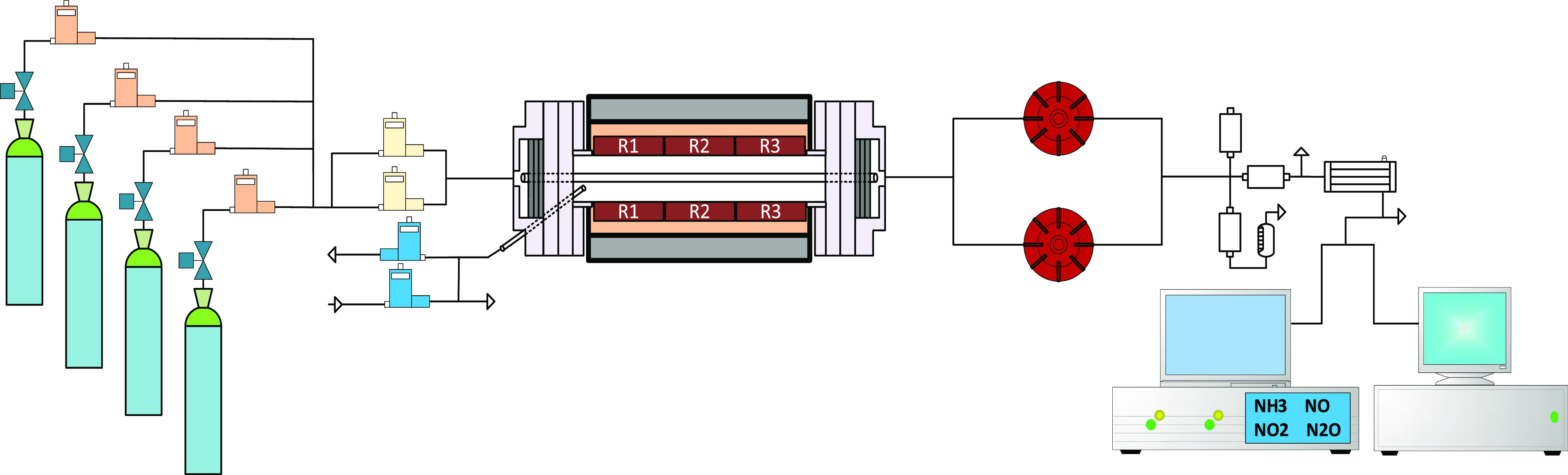
Laboratory-scale high-pressure setup.

Conversion of reactants and formed products during the combustion of a NH_3_/CH_4_ mixture are studied at high pressures (10, 20, 30, and 40 bar) under well-controlled laboratory-scale conditions. The experimental setup, which has been used in success in previous studies^3,51−53^ is schematized in Figure 1. In the present work, a temperature range of 550–1250 K and an oxygen excess ratio (λ) ranging from 0.7–3 have been considered.

**Figure 2:**
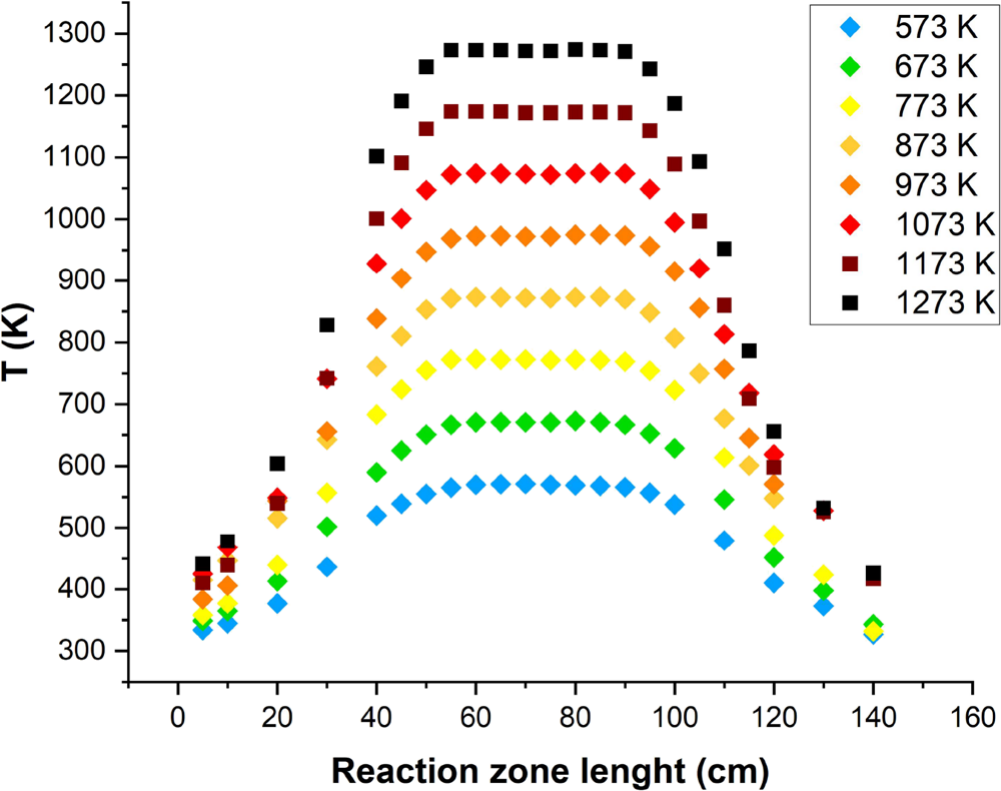
Temperature profiles at 40 bar.

The reactant gases are fed from gas cylinders (providers: Air Liquide, Praxair, or Messer) and premixed before entering the quartz flow reactor (153.8 cm long, inner diameter of 0.6 cm), which is placed inside a three-zone electrically heated oven, allowing an isothermal zone inside the tube of approximately 35 cm. This isothermal zone was determined experimentally through the temperature profiles performed at different temperatures and pressures. Figure 2 shows, as an example, the temperature profiles measured for the pressure of 40 bar, using a gas flow rate of 1000 mL (STP)/min, as has been used in all the experiments. Profiles for the different pressures and temperatures were also determined. The temperature profiles along the reactor were determined using a thermocouple positioned in the space between the quartz tube and the steel shell used to keep the pressure constant. The temperature measurement was taken each 5 cm in the central zone and each 10 cm at both sides of the reactor.

In the present work, the effect of the main variables has been analyzed: oxygen excess ratio (reducing, λ = 0.7, stoichiometric, λ = 1, and oxidizing, λ = 3, conditions), pressure (10, 20, 30, and 40 bar) and temperature (from 550 to 1250 K) and CH_4_/NH_3_ ratio (0.5, 1, and 2), which means with methane nominal concentration of 500, 1000, and 2000 ppm, respectively, for a nominal ammonia concentration of 1000 ppm.

The oxygen excess ratio (λ) is defined on the basis of the NH_3_ oxidation reaction to N_2_ (R1) (4NH_3_ + 3O_2_ ⇌ 2N_2_ + 6H_2_O) according to eq 1:
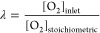
1

The flow rate is 1000 mL (STP)/min and implies a temperature- and pressure-dependent gas residence time in the isothermal reaction zone, as described in eq 2.
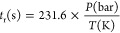
2

In the experiments, concentrations of NH_3_, CH_4_, O_2_, H_2_, NO, NO_2_, N_2_O, N_2_, CO, CO_2_, and HCN are analyzed and quantified with a gas microchromatograph (Agilent Technologies), a NH_3_/NO/NO_2_/N_2_O continuous analyzer (ABB, model: Advance Optima AO2020), a CO/CO_2_ continuous analyzer (ABB, model: Advance Optima AO2000) and a Fourier-transform infrared (FTIR) spectroscopy analyzer (Protea, model: ProtIR 204M).

The estimated uncertainty of the measurements is within ±5% but not less than 5 ppm for the continuous analyzers and 10 ppm for the gas microchromatograph and FTIR determinations. As mentioned, mixtures are diluted in argon.

Table 1 summarizes the experimental initial conditions. Sets 17, 22 and 17R, 22R correspond to repetition experiments. The experimental results of repeated experiments will be later compared to evaluate the repetitiveness of the experimental results. The influence of pressure at different temperatures has been evaluated in the 10 to 40 bar range for stoichiometries of 1 and 3 (sets 2–6, 8–15, and 17–22 in Table 1). For the highest pressure studied, 40 bar, we have also considered a fuel-rich stoichiometry of 0.7 (sets 1, 7, and 16 in Table 1), as the experimental higher pressure conditions allowed us to see the NH_3_ reaction at comparatively lower temperatures.

**Table 1:** Matrix of Experimental Conditions^a^

set	NH_3_ (ppm)	CH_4_ (ppm)	O_2_ (ppm)	*P* (bar)	λ
1	1113	560	1246	40	0.64
2	971	504	1772	20	1.02
3	936	545	1709	40	0.95
4	961	527	5234	20	2.95
5	959	507	5198	30	3.00
6	959	550	5313	40	2.92
7	1095	1094	1924	40	0.64
8	925	1085	2915	10	1.02
9	933	1136	2807	20	0.94
10	1092	1100	2646	30	0.88
11	933	1094	2705	40	0.94
12	968	1082	8385	10	2.90
13	915	1073	8456	20	2.99
14	931	1072	8185	30	2.88
15	935	1115	8255	40	2.82
16	1119	2070	3350	40	0.67
17	953	2178	4826	10	0.95
17R	938	2151	4782	10	0.96
18	937	2138	4921	20	0.99
19	983	2137	4850	40	0.97
20	928	2161	14150	20	2.82
21	1093	2055	14049	30	2.85
22	918	2097	13997	40	2.87
22R	1063	2065	14336	40	2.91

a All experiments are performed in the 550–1250 K temperature interval with a total flow rate of 1000 mL (STP)/min and using Ar as a bath gas. The residence time is defined by eq 2.

## Kinetic Modeling

The experimental results of the present work are simulated using a gas-phase chemical kinetic mechanism based on earlier work on nitrogen chemistry by Glarborg et al.,^54^ drawing on more recent work on amine chemistry by Stagni et al.,^55^ updated adding an acetonitrile reaction subset by Alzueta et al.^56^ as well as a reaction subset of methylamine by Glarborg et al.,^57^ updated by Marrodán et al.,^58^ and including modifications and/or recommendations of the recent studies of Burke,^59^ Klippenstein et al.,^60^ Marshall et al.,^61^ Alzueta et al.,^13,44^ and Glarborg et al.^62−64^ Reactions recently revised such as NH_2_ + HO_2_ by Klippenstein et al.^65^ and NH_2_ + NO_2_ by Glarborg,^64^ as well as steps involved in amine pyrolysis^61,63^ and H_2_NO reactions proposed by Stagni et al.^66^ were included as well. Other steps involved in amine chemistry are updated from the work of Cobos and Gao.^67,68^

The mechanism has been extended to include DME conversion including several reaction subsets taken from previous work of Marrodán et al.,^69^ which were tested and validated under atmospheric-pressure conditions^70^ and modified to consider high-pressure conditions by Marrodán et at.^69,71,72^ These modifications include C_1_–C_2_ and NO interactions, proposed by Glarborg et al.^73^ and have been revised and updated according to more recent studies involving NO_*x*_ that work developed and validated under high-pressure conditions,^74−78^ as well as reaction subsets for compounds such as ethanol, C_2_H_2_ proposed by Alzueta et al.^79^ for atmospheric conditions and modified by Giménez et al.^78^ to consider high-pressure conditions. The DME subset taken from the work of Alzueta et al.^80^ at atmospheric pressure has been updated according to more recent mechanisms^81,82^ to take into account the high-pressure effects. Additionally, the mechanism includes a subset for oxidation of formic acid based on the work of Marshal and Glarborg,^83^ reaction subsets for methyl formate,^71^ dimethoxymethane,^51^ and ethanol^72^ taken from different sources, as reported. The thermodynamic data come from the same sources as for the kinetic mechanisms used. The full mechanism is available as the Supporting Information.

The full compiled mechanism with the modifications mentioned has been tested, compared, and validated against several experimental data sets using different experimental set-ups in a wide range of conditions by Marrodán et al.^69^ for DME mixtures.

Calculations have been carried out in the present work using the plug-flow reactor (PFR) model of the Chemkin Pro suite (2016),^84^ the initial conditions for each experiment as listed in Table 1, and a “fix gas temperature” type problem, using the nominal reaction temperature at the flat temperature zone since similar results were obtained with and without the measured temperature profiles. The validity of the model and the mechanism has been assessed using different literature studies in which flow reactor data at atmospheric pressure were presented.^12^^,^^18^ Comparison of literature experimental results, shown in Table S1 of the Supporting Information, and calculations with the mechanism compiled and updated in this work are included in Figures S1 and S2 of the Supporting Information. The performance of the model is very good as well for the atmospheric flow reactor experimental data of Figures S1 and S2, which indicates that the model does a good job simulating the conversion of ammonia/methane mixtures, as will also be seen as follows.

## Results and Discussion

### Effect of Pressure

**Figure 3:**
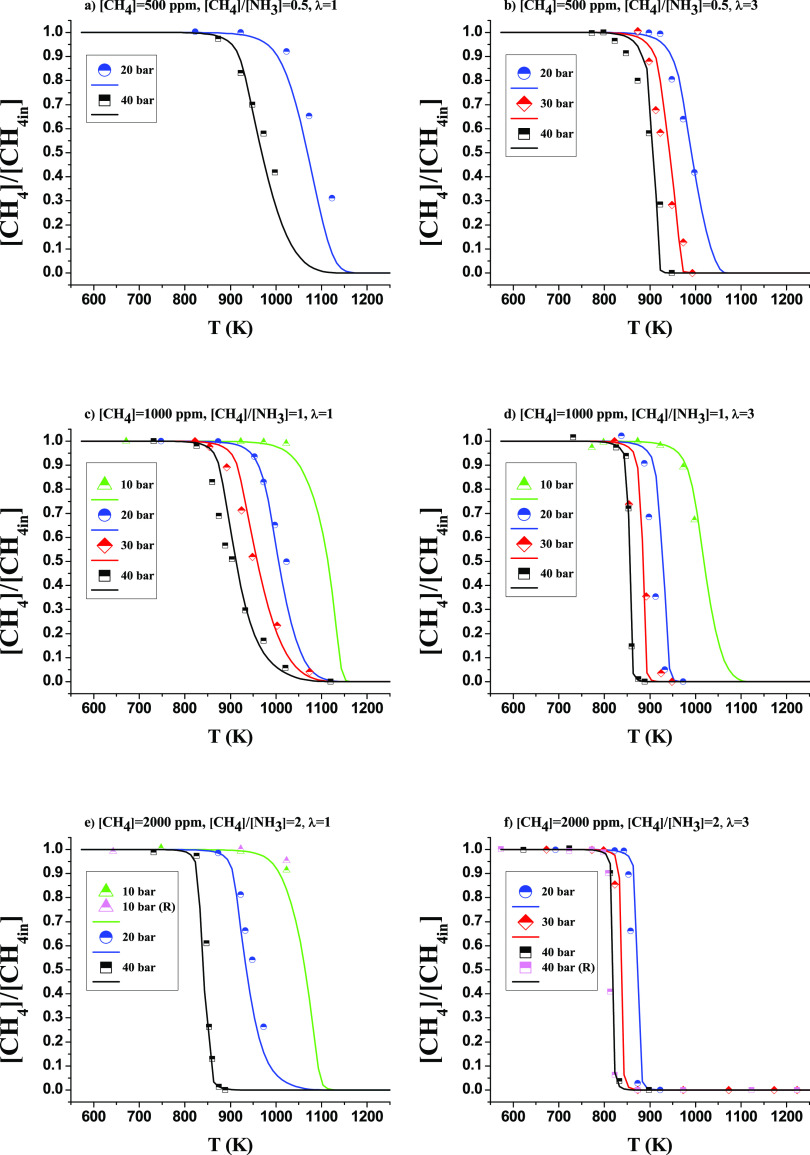
CH_4_ conversion as a function of temperature for different pressures (10–40 bar). Sets 2–6, 8–15, and 17–22 in Table 1. [CH_4_] = 500 ppm (a, b), 1000 ppm (c, d), and 2000 ppm (e, f). [NH_3_] = 1000 ppm. Left column λ = 1 and right column λ = 3. Ar as a bath gas. Symbols are used to represent experimental data and lines for calculations. Residence time is defined by eq 2.

Figure 3 shows the conversion of CH_4_ as a function of temperature for different pressures (from 10 to 40 bar), different CH_4_/NH_3_ ratios (0.5, 1, and 2), and oxygen excess ratios (λ = 1 and 3). Symbols denote experimental results and lines model calculations, from now on. As can be seen, the reaction onset temperature of CH_4_ oxidation shows a pressure dependence as also pointed out by other authors.^43^ Generally, this temperature decreases markedly with increasing temperature at all pressures studied, as also observed with pure ammonia oxidation at high pressure.^53,85^

For a pressure increase of 20 bar (from 20 to 40 bar), keeping the rest of the conditions similar, the CH_4_ oxidation reaction onset temperature decreases under stoichiometric conditions (λ = 1) is 150 K (1025 K → 875 K), 100 K (925 K → 825 K) and 50 K (875 K → 825 K) and under oxidizing conditions (λ = 3) is 75 K (900 K → 825 K), 25 K (850 K → 825 K) and 25 K (825 K → 800 K), for the CH_4_/NH_3_ ratio of 0.5, 1, and 2, respectively. The different reaction onset temperatures for both NH_3_ and CH_4_ are summarized in Table S2 of the Supporting Information. Under the current studied conditions, it is noteworthy that, for the same stoichiometry, the higher the CH_4_/NH_3_ ratio, the lower the effect of pressure on the onset temperature of the CH_4_ oxidation reaction. This effect is also observed when we switch from stoichiometric to oxidizing conditions, keeping a similar CH_4_/NH_3_ ratio. This is in line with the findings of other authors.^12,43^

The experimental results are compared to modeling predictions using the kinetic mechanism previously described. The model generally reproduces very well the trends of CH_4_ consumption, for the different pressures under the stoichiometric and oxidizing conditions of Figure 3. Modeling calculations indicate that the full conversion of CH_4_ is obtained approximately at the same temperature as those in the experimental results.

CH_4_ consumption takes place mainly through H atom abstraction by OH (R8) and by reaction with the amine radical (R9). At the highest temperatures considered, (R8) becomes the main CH_4_ consumption reaction under the studied conditions. Alzueta et al.^12^ also found (R8) and (R9) as the main CH_4_ conversion reaction in a flow reactor study of NH_3_/CH_4_ oxidation at atmospheric pressure. Under certain conditions, CH_4_/NH_3_ = 0.5, reaction (R9) is the main CH_4_ consumption reaction. This is in line with the findings of Dai et al.^43^ who suggest that (R9) becomes more important at CH_4_/NH_3_ = 0.05 than for CH_4_/NH_3_ = 0.5.
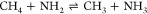
R9

Figure 3 also shows the repeatability of sets 17, 22 and 17R, 22R, respectively. As seen, the reproducibility of the experiments is very good in all the temperature ranges considered, which is an indication of the good performance of the experimental system and experimental procedure.

**Figure 4:**
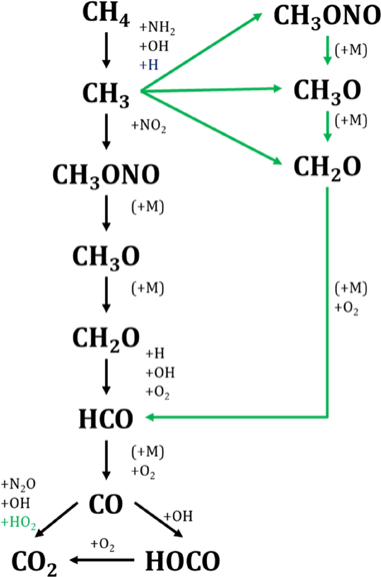
Reaction path diagram for consumption of CH_4_ at CH_4_/NH_3_ = 1 for reducing (λ = 0.7) at 40 bar, stoichiometric (λ = 1) and oxidizing (λ = 3) conditions at 10 and 40 bar. Sets 7, 8, 11, 12, and 15 on Table 1. Black lines represent the common path for the different conditions, and green lines show the additional path happening at 10 bar.

To get some insight into the reaction pathway through which the oxidation conversion of CH_4_ in the presence of NH_3_ proceeds at high pressure and different stoichiometries, we have made reaction pathway analyses for the different conditions considered. Figure 4 shows the reaction path diagram for CH_4_ consumption at [CH_4_] = 1000 ppm, and λ = 1 and 3 for the highest (40 bar) and lowest (10 bar) studied pressures when 10% of the NH_3_ is consumed. The only difference observed occurs at λ = 1 and 10 bar (green). In any case, at the end, the same species are involved, producing CO and CO_2_ as a general reactor output product.

The species and pathways marked in green are important only for 10 bar and stoichiometric conditions. As can be seen, the sequence CH_4_ → CH_3_ → CH_3_ONO → CH_3_O → CH_2_O → HCO → CO → CO_2_ is the major CH_4_ consumption channel.

**Figure 5:**
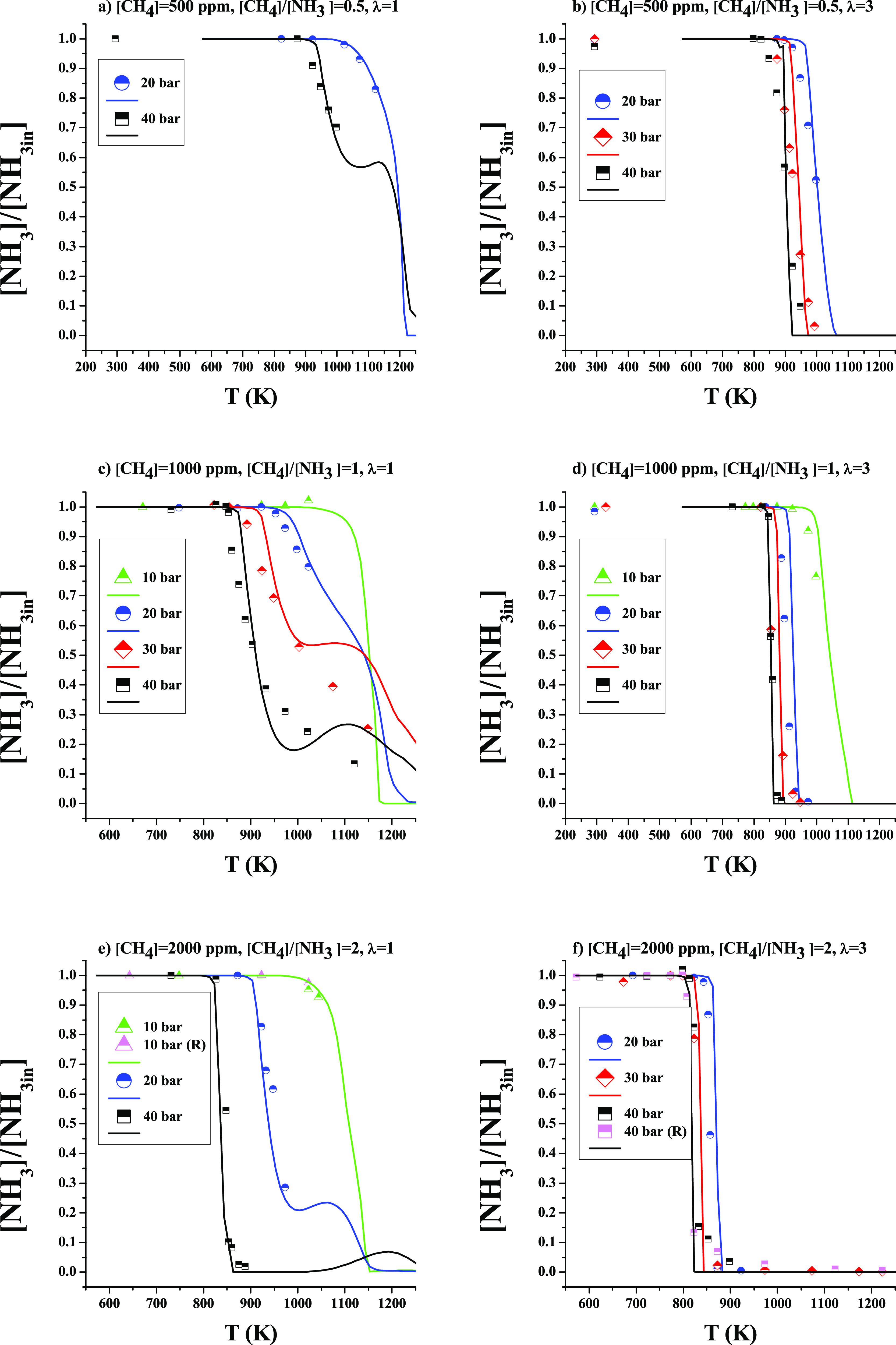
NH_3_ conversion as a function of temperature for different pressures (10–40 bar). Sets 2–6, 8–15, and 17–22 in Table 1. [CH_4_] = 500 ppm (a, b), 1000 ppm (c, d), and 2000 ppm (e, f), [NH_3_] = 1000 ppm. Left column λ = 1 and right column λ = 3. Ar as a bath gas. Symbols are used to represent experimental data and lines for calculations. Residence time is defined by eq 2.

Figure 5 shows the conversion of NH_3_ as a function of temperature for different pressures (from 10 to 40 bar), different CH_4_/NH_3_ ratios (0.5, 1 and 2) and oxygen excess ratios (λ = 1 and 3), i.e., similar conditions as Figure 3. For any pressure studied, the NH_3_ consumption shows the same reaction tendency as CH_4_ as the temperature increases. The NH_3_ concentration is sharply reduced at a given temperature. Both the NH_3_ oxidation onset temperature and the decrease of the onset temperature for NH_3_ consumption due to the pressure increase coincide with those given above for CH_4_. This effect also happens at atmospheric pressure where both species began to be consumed at the same temperature.^12^ As can be seen in Figure 5, the pressure effect on the onset reaction temperature is more pronounced under stoichiometric conditions than at oxidizing ones, contrary to what happens for pure ammonia oxidation,^53^ where the pressure increase had an effect independent of the stoichiometry. Thus, bearing in mind the use of ammonia as a fuel, its mixtures with methane provide us benefits when working with excess oxygen and increasing the pressure due to the decrease in the NH_3_ oxidation reaction onset temperature from 1100 K^53^ to 800 K for CH_4_/NH_3_ = 0 and 2, respectively, at 40 bar.

For a given stoichiometry, the pressure effect is more pronounced at lower CH_4_/NH_3_ ratios. The conditions under which an increase in pressure is least noticeable are CH_4_/NH_3_ = 2 and λ = 3 (Figure 5). In all studied cases of NH_3_ oxidation, NH_3_ starts to be consumed by reaction (R2) as well known in the literature.^12,13,53^

Figure 5 e,f, shows the repeatability of sets 17, 22 and 17R, 22R conditions, respectively. As for CH_4_, the reproducibility of the experiments is very good in all the temperature range considered.

**Figure 6:**
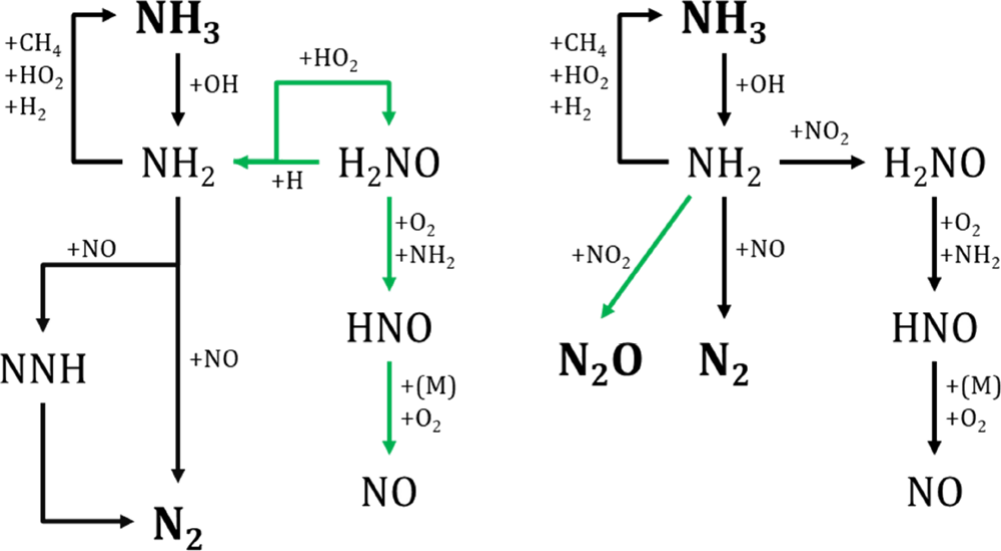
Reaction path diagram for the consumption of NH_3_ at CH_4_/NH_3_ = 1 for reducing conditions (λ = 0.7) at 40 bar and stoichiometric conditions (λ = 1) at 10 and 40 bar (left) and, oxidizing conditions (λ = 3) at 10 and 40 bar (right). Black lines represent the common path for the different conditions and green lines show the additional path happening at 10 bar (left) and 40 bar (right).

Figure 6 shows the NH_3_ consumption reaction path for CH_4_/NH_3_ = 1. On the left, for λ = 0.7 at 40 bar and λ = 1 at 10 (green) and 40 bar. On the right, for λ = 3 at 10 and 40 bar (green). In both cases, black lines represent the common path for the different conditions studied. As can be seen in Figure 6, NH_3_ → NH_2_ → H_2_NO → HNO → NO and NH_3_ → NH_2_ → N_2_ are the major consumption NH_3_ reaction channels: NO reacts with HO_2_ to form NO_2_, and with NH_2_ to form N_2_. The produced NO_2_ is quickly consumed by reaction with CH_3_ to form CH_3_ONO and with NH_2_ to form H_2_NO; thus, no appreciable quantities of this species are found at the reactor outlet.

**Figure 7:**
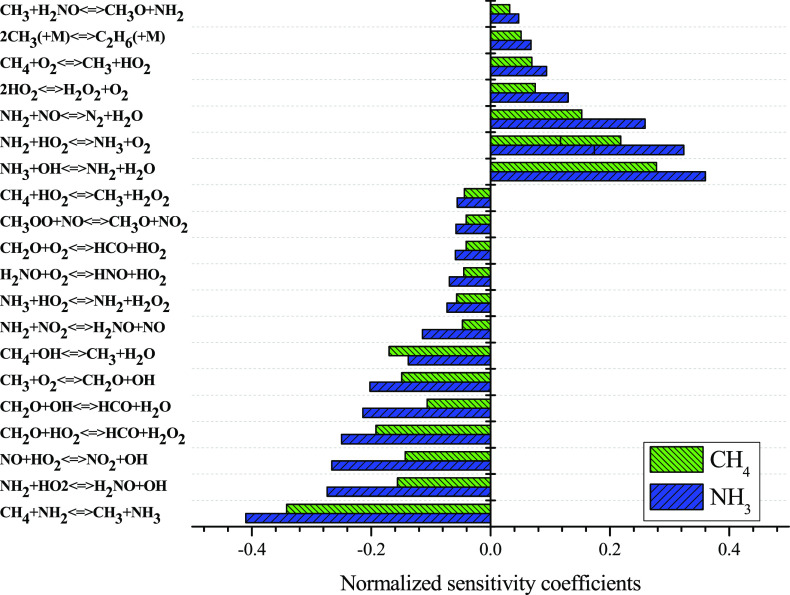
Sensitivity analysis for NH_3_ and CH_4_ conversion under the experimental conditions of Set 11 in Table 1, CH_4_/NH_3_ = 1, 40 bar, λ = 1 at 878 K.

We also performed sensitivity analysis. Figure 7 shows an example of the results obtained for CH_4_/NH_3_ = 1, at 40 bar and λ = 1 at 878 K. CH_4_ consumption is promoted by its reaction with NH_2_, OH, and HO_2_ to form CH_3_ radicals, by the reaction of CH_2_O with HO_2_, OH, and O_2_ to form HCO, by reaction of HO_2_ radical with nitrogen species (NH_3_, NH_2_, NO) to form OH radicals^43^ and by reaction of CH_3_ radicals with O_2_ also to form OH radicals. Under the same experimental conditions, the reaction of NH_3_ with OH to form NH_2_ and H_2_O, and the resulting chain reaction of NH_2_ with NO to form N_2_ and H_2_O and with HO_2_ to form NH_3_ and O_2_, together with the HO_2_ recombination to form H_2_O_2_ and O_2_,^43^ represent the major inhibition reactions for the consumption of CH_4_. In a minor extent, CH_4_ + O_2_ ⇌ CH_3_ + HO_2_ (R10) and 2CH_3_ (+M) ⇌ C_2_H_6_ (+M) (R11) appear as inhibition reactions as well. In the case of NH_3_, under the same experimental conditions, its conversion is promoted by the reaction of CH_4_ with radicals (NH_2_, OH, and HO_2_) to form CH_3_ radicals, by reactions of HO_2_ with other species (NH_2_, NO and NH_3_) to form OH radicals, by reaction of CH_3_ radicals with O_2_ to form also OH radicals, by reactions of CH_2_O with oxygenated species (OH, HO_2_ and O_2_) to produce HCO and by reaction of NH_2_ radicals with NO_2_ to form NO. As can be seen in the CH_4_ case, the consumption of NH_3_ is promoted by the production of OH, CH_3,_ and HCO species. In this case, for the inhibition of NH_3_ consumption, we find reaction (R10) CH_4_ + O_2_ ⇌ CH_3_ + HO_2_ and the recombination reactions (R11) 2CH_3_ (+M) ⇌ C_2_H_6_ (+M) and (R12) 2HO_2_ ⇌ H_2_O_2_ and the NH_3_ chain reaction of (R2) NH_3_ + OH ⇌ NH_2_ + H_2_O, followed by (R3) NH_2_ + NO ⇌ N_2_ + H_2_O or (R13) NH_2_ + HO_2_ ⇌ NH_3_ + O_2_. As with CH_4_, the inhibition of ammonia combustion is favored by reactions that produce HO_2_, H_2_O, and O_2_ species. It can be considered that as the main trends of species conversion are well captured by the model, the main reaction pathways are feasible.

The main products measured in significant amounts during the conversion of NH_3_/CH_4_ mixtures are NO, N_2_O, N_2_, H_2_, CO, and CO_2_. N_2_ and N_2_O are the most abundant nitrogen species since NO is consumed by reaction with NH_3_.^44^ NO_2_ is below 10 ppm in all studied experimental conditions, which is consistent with the results of the previous studies for pure ammonia oxidation.^53^Figures S3 and S4 of the Supporting Information compare, respectively, experimental and simulated results of N_2_ and NO obtained during the oxidation of NH_3_ in its mixtures with CH_4_ at different oxygen excess ratios for each pressure studied. NO is produced under oxidizing conditions and CH_4_/NH_3_ = 1 and 2. Model calculations reproduce well the experimental observations. NO production does not follow a clear trend with varying pressure, with NO concentration increasing with pressure for CH_4_/NH_3_ = 1 and decreasing for CH_4_/NH_3_ = 2 with a similar pressure variation (Figure S4). Also, the difference in the concentration of NO produced is not remarkable. For the same pressure, NO production is favored by CH_4_ addition, with a peak of 16 ppm for CH_4_/NH_3_ = 1 and 36 ppm for CH_4_/NH_3_ = 2. Probably, the diminution of the NH_3_ ratio in the mixture provokes this NO increase in the exhaust gases,^28^ which is a drawback of using mixtures with high CH_4_/NH_3_ ratios. The main production reaction of NO is (R14) and to a minor extent (R15), (R16) and (R17). It is noted that (R15) and (R16) are more noticeable for λ = 3 than λ = 1, and (R17) is only remarkable at the highest pressures.
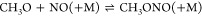
R14
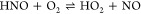
R15
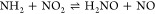
R16
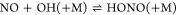
R17

NO is consumed mainly through reaction (R18) to form NO_2_ and to a minor extent via reactions (R3) and (R4) to form N_2_ and NNH.
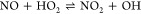
R18

This is consistent with the results obtained in our previous work on high-pressure ammonia consumption in a flow reactor^53^ in which NO was not produced under stoichiometric conditions. The typical thermal DeNO_*x*_ reaction NH_2_ + NO ⇌ NNH + OH has not been found to be very important under the studied conditions, as other authors have.^20^

**Figure 8:**
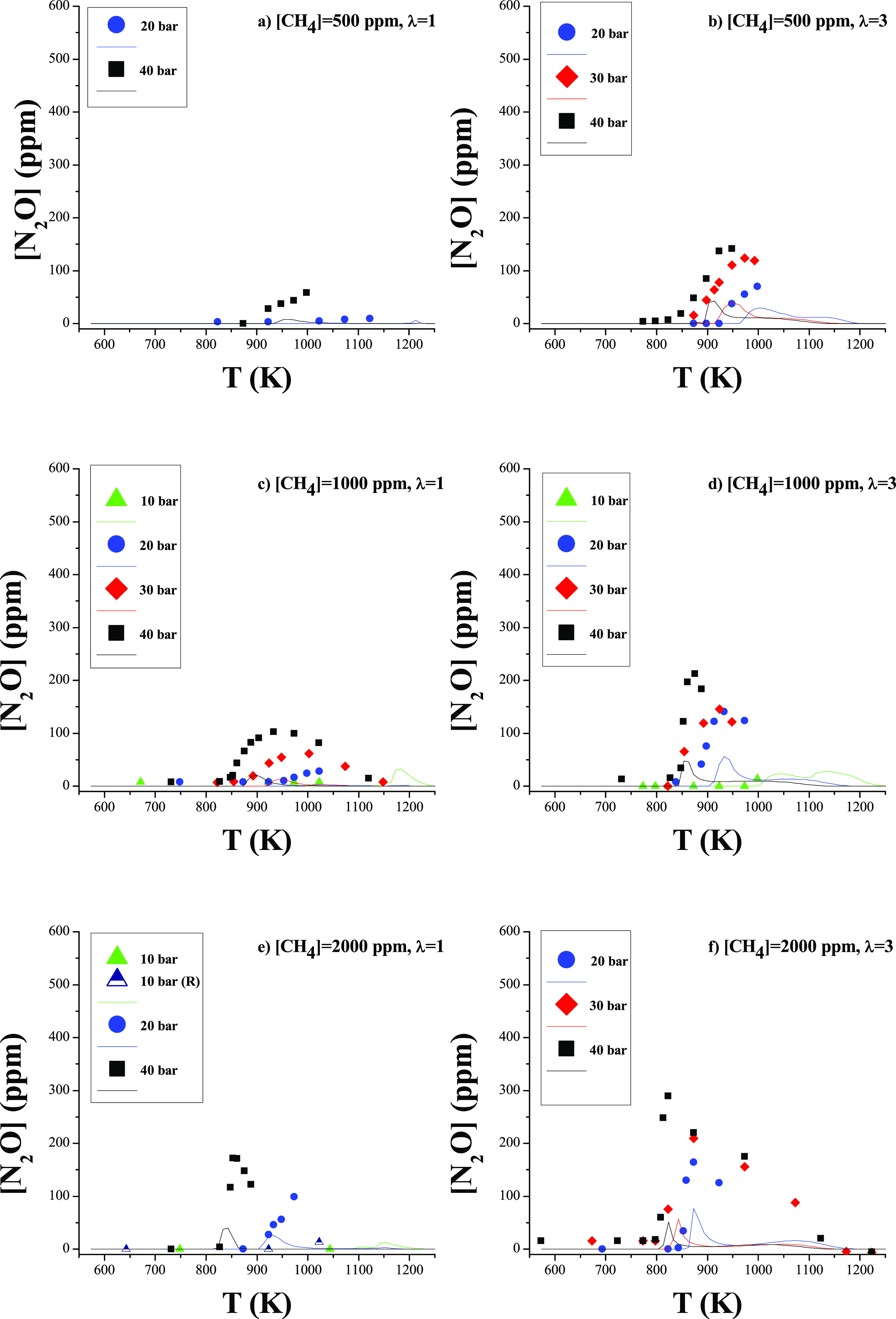
N_2_O production as a function of temperature for different pressures (10–40 bar). Sets 2–6, 8–15 and 17–22 in Table 1. [CH_4_] = 500 ppm (a, b), 1000 ppm (c, d), and 2000 ppm (e, f). [NH_3_] = 1000 ppm. Left column λ = 1 and right column λ = 3. Ar as a bath gas. Symbols are used to represent experimental data, and lines for calculations. Residence time is defined by eq 2.

In contrast, we found a clear effect of pressure on the N_2_O concentration at the reactor output, which consists of obtaining a higher N_2_O concentration at higher working pressures. A direct relationship has also been found between the CH_4_/NH_3_ ratio and the N_2_O production, which will be discussed below. Figure 8 (rescaled as Figure S5 in the Supporting Information) shows the results of N_2_O concentration as a function of temperature. In that figure, a maximum of 140, 215, and 290 ppm of N_2_O at 40 bar at λ = 3 for CH_4_/NH_3_ ratios of 0.5, 1, and 2, respectively is observed. Model calculations reproduce the main trends observed experimentally, even though they underpredict the specific values. The model simulations indicated that the N_2_O concentration is very low under all studied conditions, but, as can be seen in Figure 8, this does not occur experimentally, where concentrations are higher than calculations. According to the model, the production of N_2_O takes place through reactions (R19) and (R20), in comparison to atmospheric pressure where this occurs exclusively via (R20).^18^
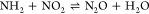
R19
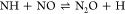
R20

In the presence of CH_4_ with high concentration levels of CO, N_2_O consumption mainly occurs through reaction (R21) as found by Sun et al.^20^ and to a minor extent through reaction.
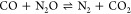
R21
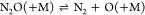
R22

Concerning N_2_, this species is produced by reaction (R3) and to a minor extent by reaction (R5). It is noticeable that at intermediates and high temperatures, in which the NH_3_ concentration is below 50%, (R21) starts to be important in N_2_ production.

**Figure 9:**
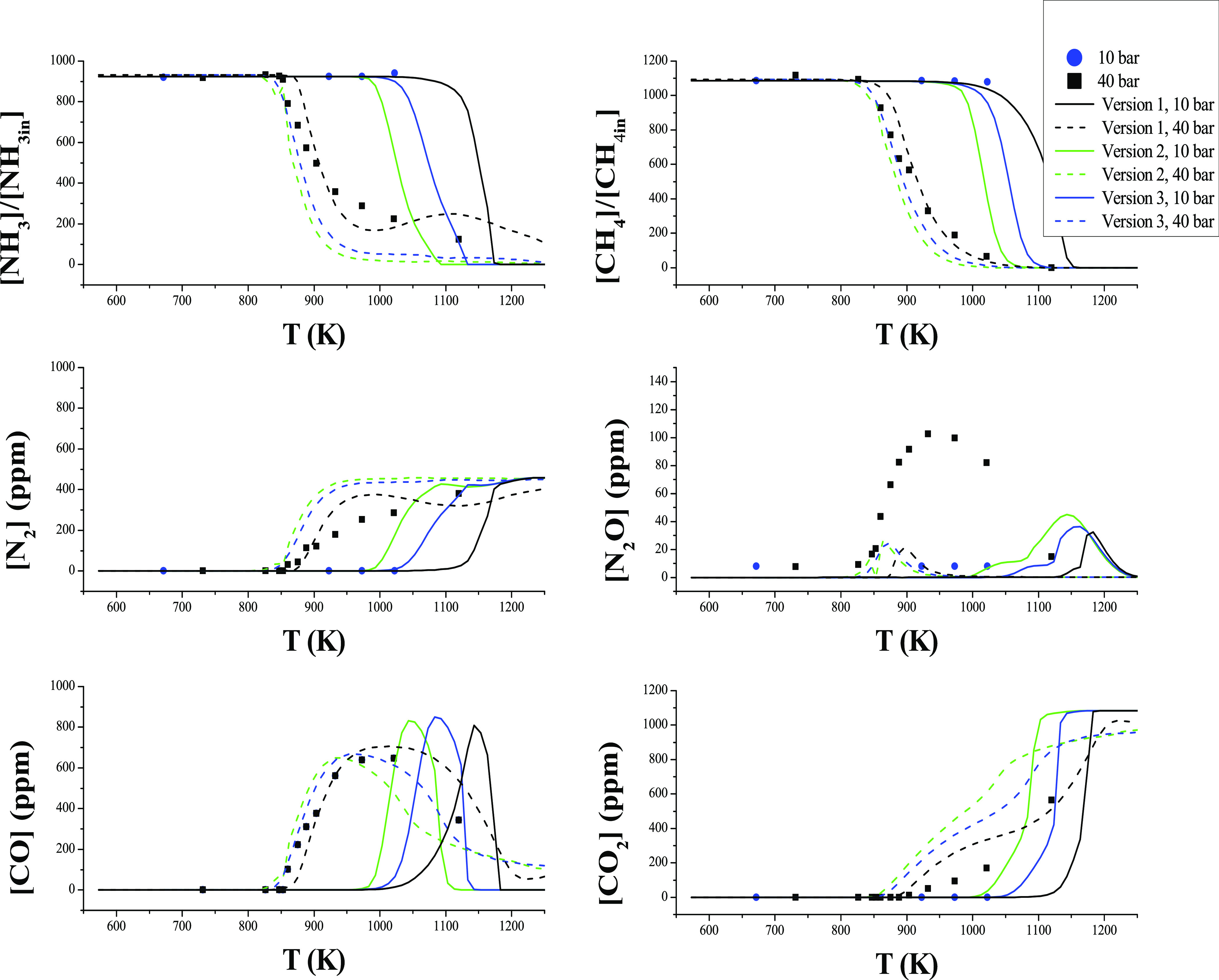
Experimental (symbols) and simulated (lines) results as a function of temperature for different pressures (10–40 bar) and λ = 1. Sets 8–11 in Table 1. [CH_4_] = 1000 ppm [NH_3_] = 1000 ppm. Ar as a bath gas. Residence time is defined by eq 2.

Regarding CO, in Figure 9, CO is produced through reactions (R23) and (R24) under all studied conditions. Reaction (R24) is favored by the increase in pressure, which is even more noticeable under oxidizing conditions. Also, as the CH_4_/NH_3_ ratio increases, this effect is accentuated, with the (R23) reaction being negligible at CH_4_/NH_3_ = 2.
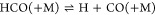
R23
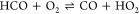
R24

CO is then consumed to produce CO_2_ by (R21), (R25) and (R26) reactions and to produce HOCO, via reaction (R21), which is then converted to CO_2_ by oxidation (R28).
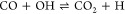
R25
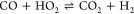
R26
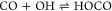
R27
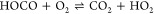
R28

As far as consumption reactions are concerned, while (R25) is always present, (R26) is important only for the lowest pressure studied and (R27) and (R28) for the higher pressure conditions considered.

We have evidenced some instability in calculations with the model under the given conditions. This issue is more relevant as the pressure increases, in particular, for stoichiometric conditions. In order to analyze this in certain detail, we performed some tests. It is found that the reaction NH_2_ + HO_2_ → products is a key step as pointed out^43,85^ and the possible product channels of this reaction are (R13), (R29), and (R30). Therefore, optimizing the mechanism of NH_3_ conversion for high pressure needs to take into account the rate constant of H_2_NO-related reactions, which have been reported to be relevant.^85^ At present, we do not know the reason for the discrepancy between the experimental and calculation results, but this is an aspect that deserves further specific studies.
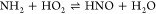
R29
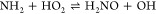
R30

Figure 9 shows the experimental and simulation results of consumption and production of the species suffering from these instabilities (i.e., NH_3_, N_2_, and CO) with three different modifications of the current mechanism used. First, the mechanism of the present work using the reaction rates constants proposed by Klippenstein et al.^65^ for reactions (R13), (R29), and (R30) without modifications (version 1 of the mechanism). Then, we have used two other versions of the mechanism: one using the rate constants of Sumathi et al.^86^ for reactions (R13), (R29), and (R30) (version 2 of the mechanism), and the same mechanism of version 1, in which reaction (R30) has been multiplied by 10 (version 3 of the mechanism). With both versions 2 and 3 of the mechanism, instabilities disappear (Figure 9), and the changes made in version 3 resulted in a large overprediction of N_2_ and CO_2_. Thus, we can state that the most critical reaction when it comes to produce instabilities is reaction (R30), where the rate value appears to be critical for the model performance.

The instability issue may also be attributed to thermochemical data of participating species, mainly HNO, or to the evolution of the H_2_NO species which has been identified as important in other ammonia studies.^54^

As seen in Figure 9, the best match of the experimental results and calculations made with the 3 versions of the mechanism is obtained with version 3. As has been mentioned, multiplying the rate constant of reaction (R30) by 10 acts to avoid the instabilities happening with the mechanism proposed in the present work, i.e., version 1, which includes without changes the rate constant proposed by Klippenstein et al.^65^ Version 2 of the mechanism includes the rate constants of Sumathi et al.^86^ for reactions (R13), (R29), and (R30), and also avoids instabilities, even though it largely overpredicts the conversion of NH_3_ and production of N_2_ and CO_2_. For the N_2_O production cases, none of the 3 versions solve the problem of instabilities. Additionally, it has to be noted that the recent work by Klippenstein and Glarborg on the reaction rate of NH_2_ + HO_2_ is probably more accurate than the factor of 10 used in version 3 of the mechanism, as necessary to minimize instabilities, since that determination would exhibit an uncertainty lower than an order of magnitude. However, the use of the 3 modified versions of the mechanism does intend to show that the model still needs improvement, and more work on this is desirable. A possible way for improvement may rely on the formation of C/N species, such as methylamine or a higher amount of nitromethane, whose description may also need refinement. A hypothetical formation of PAH may also happen, in particular under fuel-rich conditions.

As discussed in previous work by our group,^3,51,53,58,69,71,72,87^ it is important to bear in mind that the changing in pressure in our experimental system and procedure, implies the change of the residence time according to eq 2, as has been discussed and analyzed in earlier studies.^53,69,87^ In these studies, it was concluded that both pressure and residence time simultaneously affected the formation and concentration of products. Compared to the work of Marrodán et al.,^69,87^ in the present results, pressure has a major influence on the methane and ammonia conversion, as can be seen in Figures S6 and S7 of the Supporting Information.

### Effect of the Oxygen Excess Ratio

**Figure 10:**
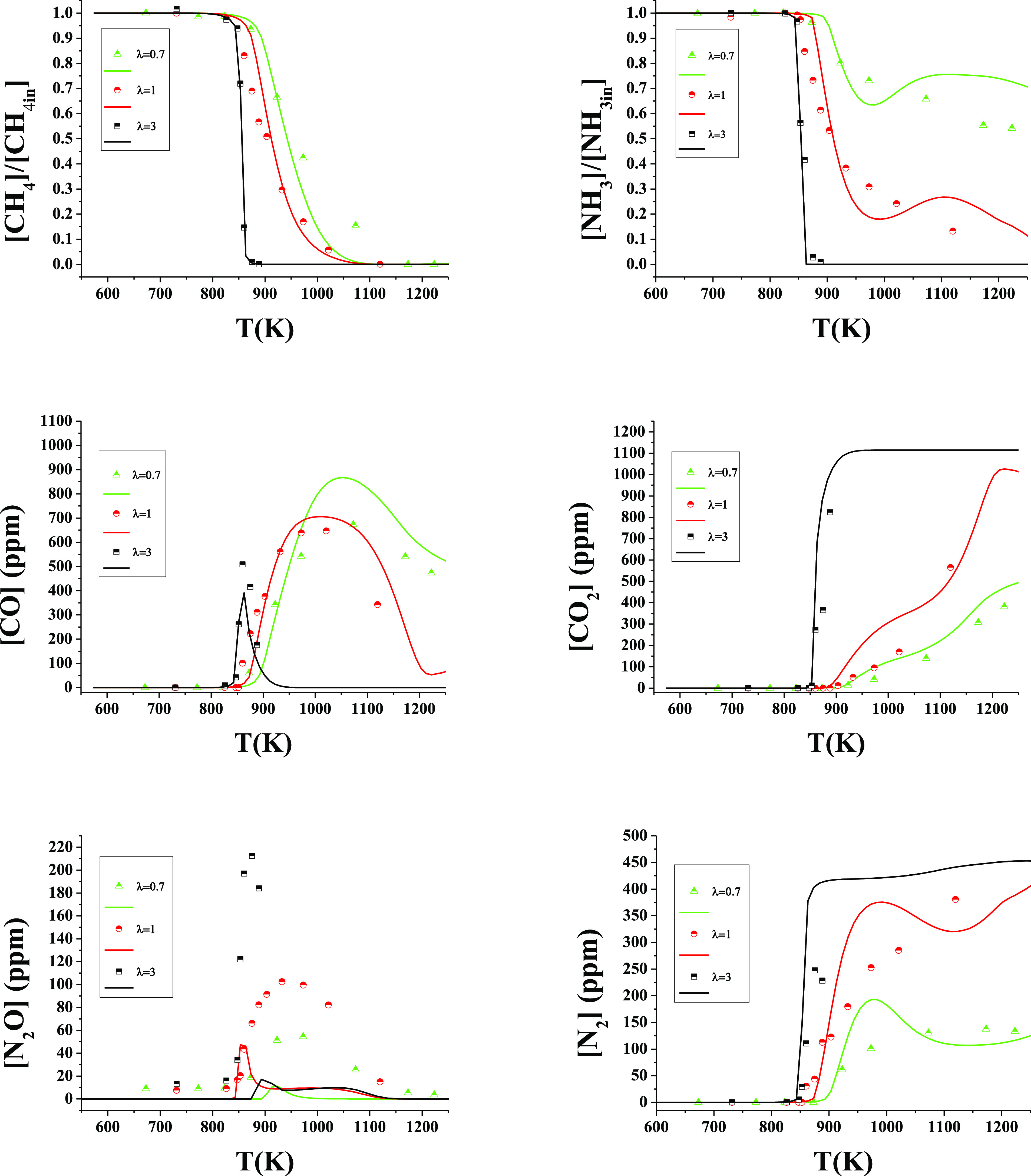
Conversion of NH_3_, CH_4_, CO, CO_2_, N_2_O and N_2_ as a function of temperature at 40 bar and different stoichiometries. Ar as a bath gas. Sets 7, 11, and 15 in Table 1. (λ = 0.64:1095 ppm of NH_3_, 1094 ppm of CH_4_; λ = 0.94:933 ppm of NH_3_, 1094 ppm of CH_4_; λ = 2.82:935 ppm of NH_3_, 1115 ppm of CH_4_).

Figure 10 includes the results of varying the oxygen excess ratio (λ = 0.7, 1 and 3) at 40 bar for CH_4_/NH_3_ = 1, to show the effect of the availability of O_2_ availability. Experimental results show a clear sensitivity to oxygen availability. Reactant conversion is shifted to lower temperatures when the O_2_ concentration is increased. Full conversion, where possible under the conditions studied, is also achieved at lower temperatures and higher oxygen concentrations. As the working pressure decreases, the decrease in reaction onset temperature is more pronounced, and the same effect occurs at lower CH_4_/NH_3_ ratios. Switching from stoichiometric to oxidizing conditions, at 20 bar, the reaction onset temperature reduction is 125, 75, and 50 K, for CH_4_/NH_3_ = 0.5, 1, and 2. Changing from λ = 0.7 to 1 produces only a shift (50 K) for CH_4_/NH_3_ = 0.5 and 1. In the case of 40 bar and at CH_4_/NH_3_ = 1 (Figure 10), a shift to a lower temperature of the onset of the CH_4_ and NH_3_ conversion reaction has been found (50 K, 875 → 825) when moving from reducing to oxidizing conditions. In previous work of our group, it was found that the conversion of pure ammonia happens at approximately 20–25 K^53^ less under oxidizing conditions compared to stoichiometric conditions. However, when CH_4_ is added, this difference increases, as seen in the present results. Methane is mainly consumed by H-abstraction-producing CH_3_ radicals, which leads to the increase of the O/H radical pool. Also, the addition of methane leads to the production of CH_3_, CH_2_O, and HCO, species that promote ammonia consumption, as can be seen in the results of the sensitivity analysis of Figure 7.

The maximum peak of CO emissions is reached at lower temperatures and in lower concentrations with increasing excess oxygen ratio, which logically promotes full CO conversion; therefore, CO_2_ production is shifted to lower temperatures. From an environmental point of view, it is remarkable that the increase in pressure leads to an increase in the maximum N_2_O concentration observed, as pointed out before, increasing it by a factor of more than 2 when switching from stoichiometric to oxidizing conditions.

**Figure 11:**
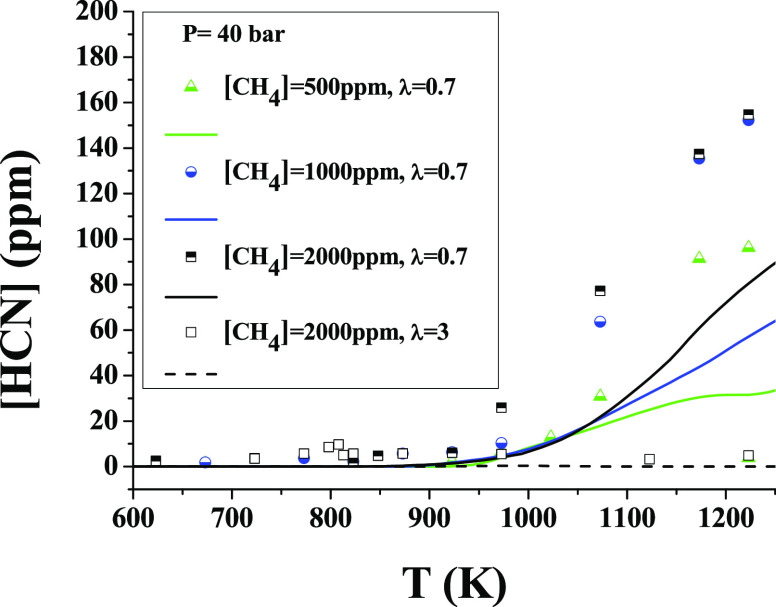
HCN conversion as a function of the temperature at 40 bar and different stoichiometries. Ar as a bath gas. Sets 1, 7, 16, and 22 are shown in Table 1 (λ = 0.64:1113 ppm of NH_3_, 560 ppm of CH_4_; λ = 0.64:1095 ppm of NH_3_, 1094 ppm of CH_4_; λ = 0.67:1119 ppm of NH_3_, 2070 ppm of CH_4_; λ = 2.87:918 ppm of NH_3_, 2097 ppm of CH_4_).

One key consideration to bear in mind when discussing a deficiency or excess of oxygen is the production of HCN. In our experimental system, under the studied conditions, it has been found that HCN is only produced under reducing conditions. Under these conditions, the HCN concentration at the outlet is higher as the proportion of CH_4_ is higher in the mixture. Figure 11 includes the results of HCN production varying the oxygen excess ratio (λ = 0.7 and 3) at 40 bar for CH_4_/NH_3_ = 2 (the most favorable CH_4_/NH_3_ ratio for HCN formation) and varying the CH_4_/NH_3_ ratio under reducing conditions, to show the effect of both O_2_ availability and CH_4_/NH_3_ ratio. Results show that, for λ = 0.7, an appreciable formation of HCN happens during the interaction between NH_3_ and CH_4_, in particular, above 1000 K when significant conversion of CH_4_ occurs. The model underpredicts the concentration of HCN by a factor of 2, even though the main trends are reasonably well captured. However, no significant formation of HCN is found for fuel-lean conditions, λ= 3.

### Effects of the CH_4_/NH_3_ Ratio

From a practical point of view, it is interesting to evaluate different CH_4_/NH_3_ ratios in order to assess the use of possible different mixtures containing both ammonia and methane.

**Figure 12:**
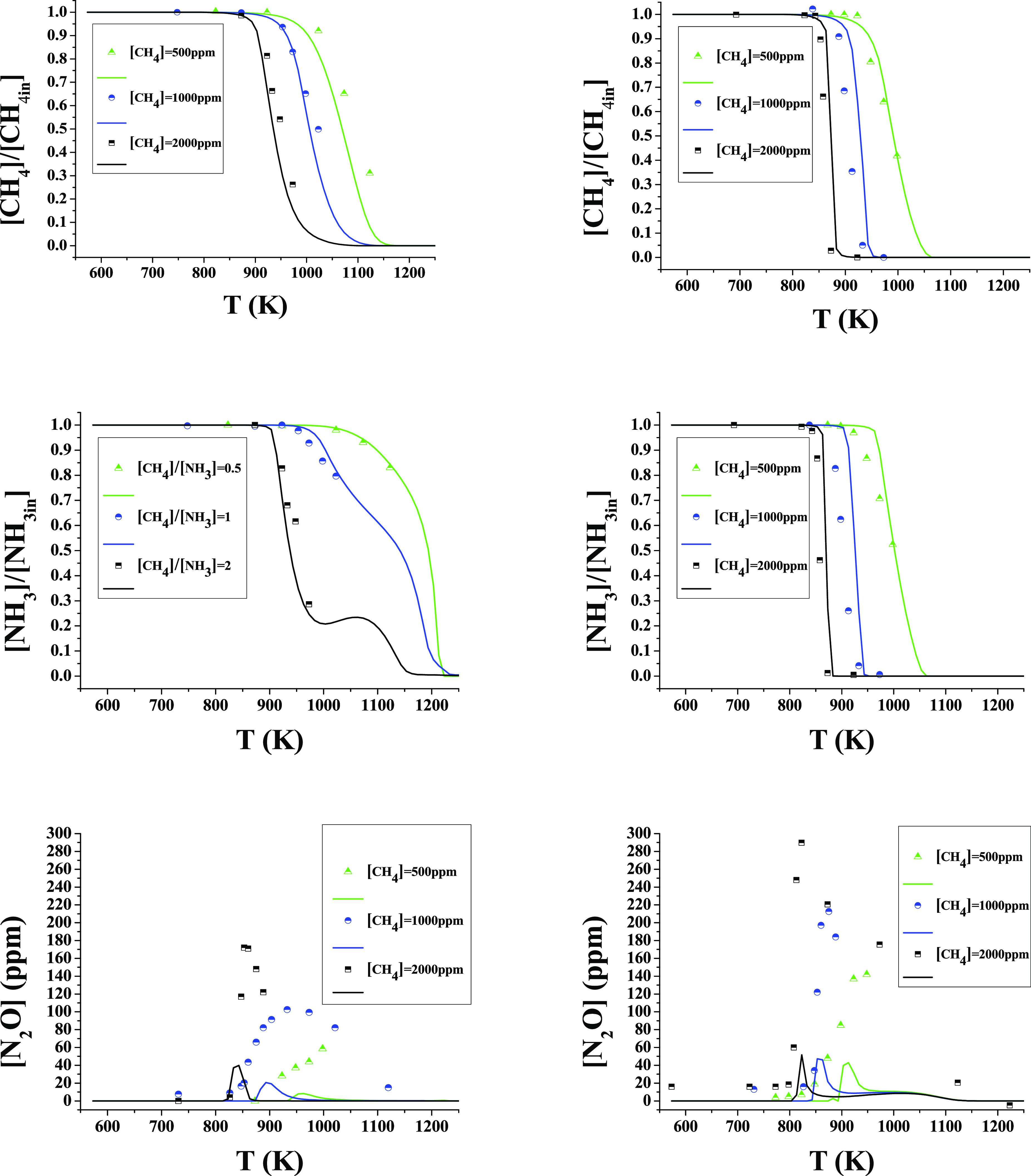
Conversion of NH_3_, CH_4_, CO, CO_2_, N_2_O, and N_2_ at 40 bar of pressure as a function of temperature and for different CH_4_/NH_3_ ratios (0.5, 1 and 3). Ar as a bath gas. Sets 3, 6, 11, 15, 19, and 22. Left column λ = 1 and right column λ = 3.

The consumption and production of species during the oxidation of CH_4_/NH_3_ mixtures are also measured as a function of the CH_4_/NH_3_ ratio, at different oxygen excess ratios (λ = 1 and 3), at 40 bar as an example. To give an idea of the CH_4_ addition effect, Figure 12 shows the consumption of CH_4_, NH_3_, and the production of N_2_O. As seen in Figure 12, for a given pressure and oxygen excess ratio, the addition of CH_4_ always results in a decrease of the reaction onset temperature of both NH_3_ and CH_4_ oxidation. This effect is more noticeable for stoichiometric conditions than for oxidizing ones. This fact is of interest, from a practical point of view, because it appears that the addition of carbon combustibles helps to diminish the ignition temperature of ammonia. For the highest pressure studied, 40 bar, pure ammonia starts to convert at approximately 1165 K under the same experimental conditions and [NH_3_] = 1000 ppm. In Figure 12, it can be seen that the onset temperature for NH_3_ conversion decreases (875 K, 825 K, 825 K, at 40 bar and CH_4_/NH_3_ = 0.5, 1, and 2, respectively) when NH_3_ is oxidized in the presence of CH_4_, for all studied CH_4_/NH_3_ ratios and stoichiometries. This is due to the increased production of OH radicals and CH_3_ radicals, which will subsequently produce more OH radicals, as discussed above. On the other hand, the major disadvantage is the production of NO and N_2_O occurs at high CH_4_/NH_3_ ratios. As seen above, the combustion of NH_3_ and CH_4_ under the studied conditions produces CH_3_ONO which decomposes into NO, which reacts to form NO_2_ at high pressure, and this later produces N_2_O. According to the mechanism, under the present conditions, the increase in N_2_O was not found to come from the production of HCN oxidation, as found by other authors,^88,89^ even though the specific operating conditions considered are different. Anyway, HCN production is not important under the conditions in which N_2_O is formed. In this sense, working with CH_4_/NH_3_ ratios higher than 1 is not desirable because it does not offer great benefits compared to CH_4_/NH_3_ ratios of 1 or 0.5, while it leads to a considerable increase of N_2_O emitted, which is a greenhouse gas with 273 times more global warming potential than that of CO_2_.^90^ Furthermore, a high CH_4_/NH_3_ concentration leads to increased emissions of CO_2_.

### Mass Balances

**Figure 13:**
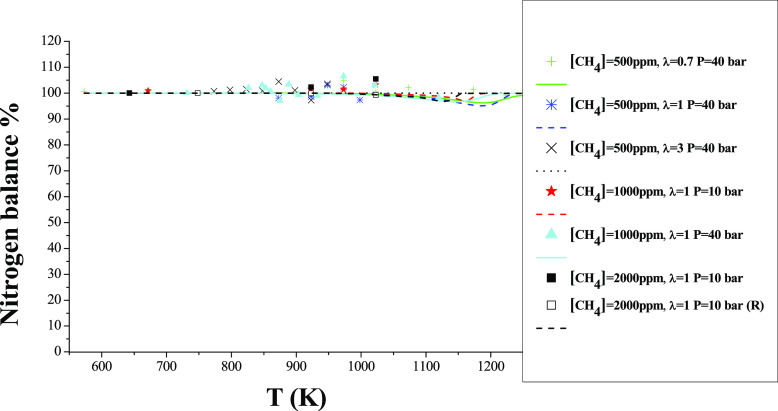
Experimental and calculated N balance during the oxidation of NH_3_, as a function of the reactor temperature, for different oxygen excess ratios, compositions, and pressures. Species included in the balance are NH_3_, NO, NO_2_, N_2_O, HCN, and N_2_. Sets 1, 3, 6, 8, 11, 22, and 22R are shown in Table 1.

In order to evaluate the quality of our experiments and to determine if the measured species are dominant under the studied conditions, we decided to do nitrogen balances for the experiments performed. We can do that because we have used argon as a bath gas, allowing in this way the precise determination of N_2_ as a product gas. Figure 13 shows, as an example, a nitrogen atom balance for different experimental conditions. The N balance is calculated by considering the nitrogen atoms of the following species: NH_3_, N_2_, HCN, NO, N_2_O, and NO_2_. Even though NO_2_ has been accounted for in the nitrogen balance determined, their experiment profiles have not been shown in the figures because these species are around the uncertainty of the equipment measurements lower than 5 ppm in all cases. The N balance (in percentage) calculated with the model considering the same species mentioned above is also shown in Figure 13 as a continuous line. As seen, the calculated N balance is between 90 and 100%, while the experimental one closes between 90 and 105% along the whole temperature range. This indicates a reasonable agreement between species determined and calculated, even though a small mass of other species not analyzed experimentally may also be present.

**Figure 14:**
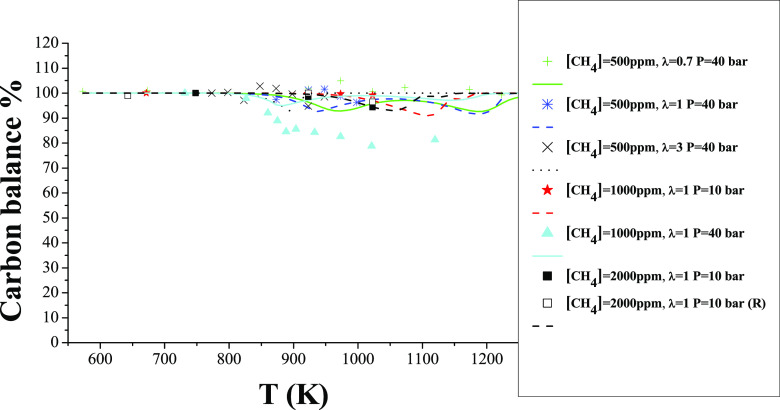
Experimental and calculated C balance during the oxidation of CH_4_, as a function of the reactor temperature, for different oxygen excess ratios, compositions, and pressures. Species included in the balance are CH_4_, CO, HCN, and CO_2_. Sets 1, 3, 6, 8, 11, 22, and 22R in Table 1.

Similarly, Figure 14 shows an example of a carbon atom balance for the same experiments shown in Figure 13. The C balance is calculated considering the carbon atoms of the following species: CH_4_, CO, HCN, and CO_2_. The C balance (in percentage) calculated with the model of the species mentioned above is also shown in Figure 14 as a line, and it is between 85 and 100%, while the experimental one closes between 90 and 105% for all studied temperatures. Results indicate a reasonable closing of the C balance as well.

## Conclusions

An experimental and simulation study of the main features of the oxidation of mixtures of ammonia with methane at high pressure (from 10 to 40 bar), under reducing, stoichiometric, and oxidizing conditions in the 550–1250 K temperature interval, in a quartz tubular flow reactor with, roughly, 1000 ppm of inlet NH_3_ and 500, 1000, and 2000 ppm of CH_4_, in NH_3_/CH_4_ mixture, and using argon as a diluent, has been performed.

The main product of ammonia conversion is N_2_, followed by N_2_O, and under certain conditions NO (CH_4_/NH_3_ = 1 and 3 at λ = 3) while the NO_2_ concentration is negligible and below the uncertainty of the measurements under all conditions studied. The use of high pressure acts to favor the formation of N_2_ and CO_2_ from ammonia oxidation compared to what happens at atmospheric pressure. This is a positive outcome for the use of ammonia as a fuel in pressure applications such as turbines. However, the N_2_O concentration in the exhaust gases is significantly higher than that in pure NH_3_ oxidation, which may be a drawback.

The onset of both NH_3_ and CH_4_ oxidation occurs at higher temperatures for reducing and stoichiometric conditions than for oxidizing conditions for all the pressures considered, indicating the importance of oxygen stoichiometry for ammonia conversion. In addition, working at higher oxygen excess ratios minimizes HCN production.

Compared to the oxidation of pure ammonia, the presence of CH_4_ acts to shift ammonia conversion to lower temperatures, up to 300 K under certain conditions.

Pressure is seen to have an important influence on both the NH_3_ and CH_4_ oxidation regimes in the mixture, shifting them to lower temperatures as the pressure increases. However, the influence of pressure is seen to be significantly more important at low pressures compared to high pressures, and, at the same time, this influence is lower at higher CH_4_/NH_3_ ratios.

The production of OH, CH_3_, and HCO species promotes the NH_3_ and CH_4_ consumption and, on the counterpart, the inhibition of NH_3_ and CH_4_ combustion is favored by reactions that produce HO_2_, H_2_O, and O_2_ species, under the studied conditions.

The mechanism compiled from the literature and updated in the present work, used to carry out the simulations, is able to describe well the conversion of both NH_3_ and CH_4_ under almost all of the studied conditions. Nevertheless, discrepancies, mainly in the prediction of NH_3_, NO, N_2_, N_2_O, and CO_2_ under certain experimental conditions, have been observed, with higher discrepancies between the experimental and calculated results seen for N_2_O. The best agreement between experimental results and calculations is found for oxidizing conditions.

## Supporting Information Available

Validation of kinetic mechanism; table of experimental onset reaction NH_3_ and CH_4_ temperatures; N_2_ and NO species concentration figures; comparison of pressure and residence time effect; and N_2_O species concentration rescaled figure (PDF)
Mechanism details (TXT)
Thermodynamic data listing (TXT)
