# Supplementary material for: Experimental and Modeling High-Pressure Study of Ammonia–Methane Oxidation in a Flow Reactor
Source: Energy Fuels. 2024 Jan 8;38(2):1399–415. doi: 10.1021/acs.energyfuels.3c03959 (PMC10804275; doi:10.1021/acs.energyfuels.3c03959)
Supplement: Supplementary file 1 — ef3c03959_si_001.pdf [file ef3c03959_si_001.pdf]

Supporting information

Experimental and modeling high-pressure study of ammonia-methane oxidation in a flow reactor

*Pedro García-Ruiz, Iris Salas, Eva Casanova, Rafael Bilbao and María U. Alzueta**

Aragón Institute of Engineering Research (I3A), Department of Chemical and Environmental Engineering, University of Zaragoza, 50018 Zaragoza, Spain

*uxue@unizar.es

Table S1. Experimental conditions for selected literature flow reactor data.

Set	CH_4 (ppm)	O_2 (ppm)	NH_3 (ppm)	NO (ppm)	Bath gas	λ	Reference
1	1063	2137	956	1020	99.47% Ar	0.70	Alzueta et al. [12]
2	1020	2770	1019	1012	99.42% Ar	0.99	Alzueta et al. [12]
3	2513	5036	468	0	99.2% N_2	0.94	Mendiara et al. [18]

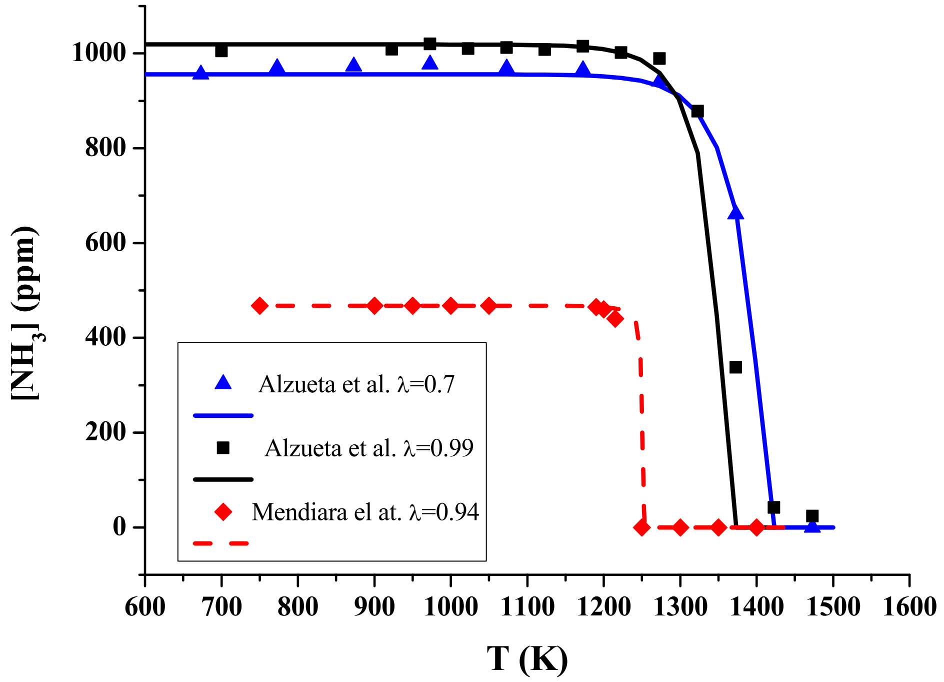

Figure S1: NH_3 conversion as a function of temperature at atmospheric pressure. Sets 1-3 in Table S1. Symbols are used to represent experimental data and lines for predictions.

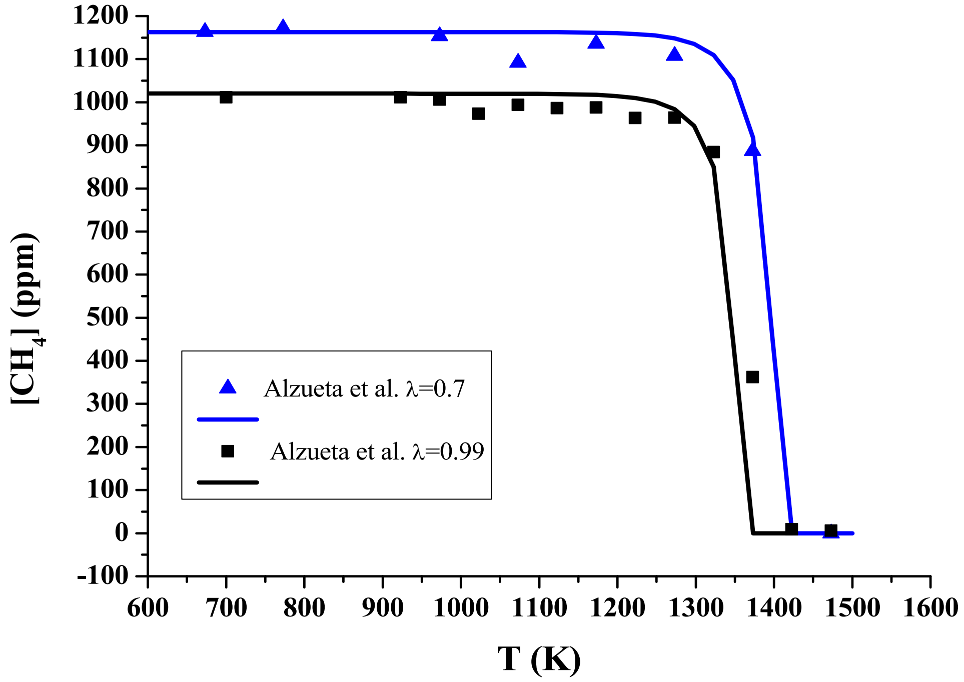

Figure S2: CH_4 conversion as a function of temperature at atmospheric pressure. Sets 1-2 in Table S1. Symbols are used to represent experimental data and lines for predictions.

Table S2. Experimental onset temperature for NH_3 and CH_4 combustion.

Initial conditions				Onset temperature	
**NH_3 (ppm)**	**CH_4 (ppm)**	**λ**	**P (bar)**	**NH_3 T (K)**	**CH_4 T (K)**
1000	500	0.7	40	925	925
1000	500	1	20	1025	1025
1000	500	1	40	875	875
1000	500	3	20	900	900
1000	500	3	30	850	850
1000	500	3	40	825	825
1000	1000	0.7	40	825	875
1000	1000	1	10	No reaction	No reaction
1000	1000	1	20	925	925
1000	1000	1	30	850	850
1000	1000	1	40	850	825
1000	1000	3	10	925	925
1000	1000	3	20	850	850
1000	1000	3	30	825	825
1000	1000	3	40	825	825
1000	2000	0.7	40	825	825
1000	2000	1	10	1025	1025
1000	2000	1	20	875	875
1000	2000	1	40	825	825
1000	2000	3	20	825	825
1000	2000	3	30	800	800
1000	2000	3	40	800	800

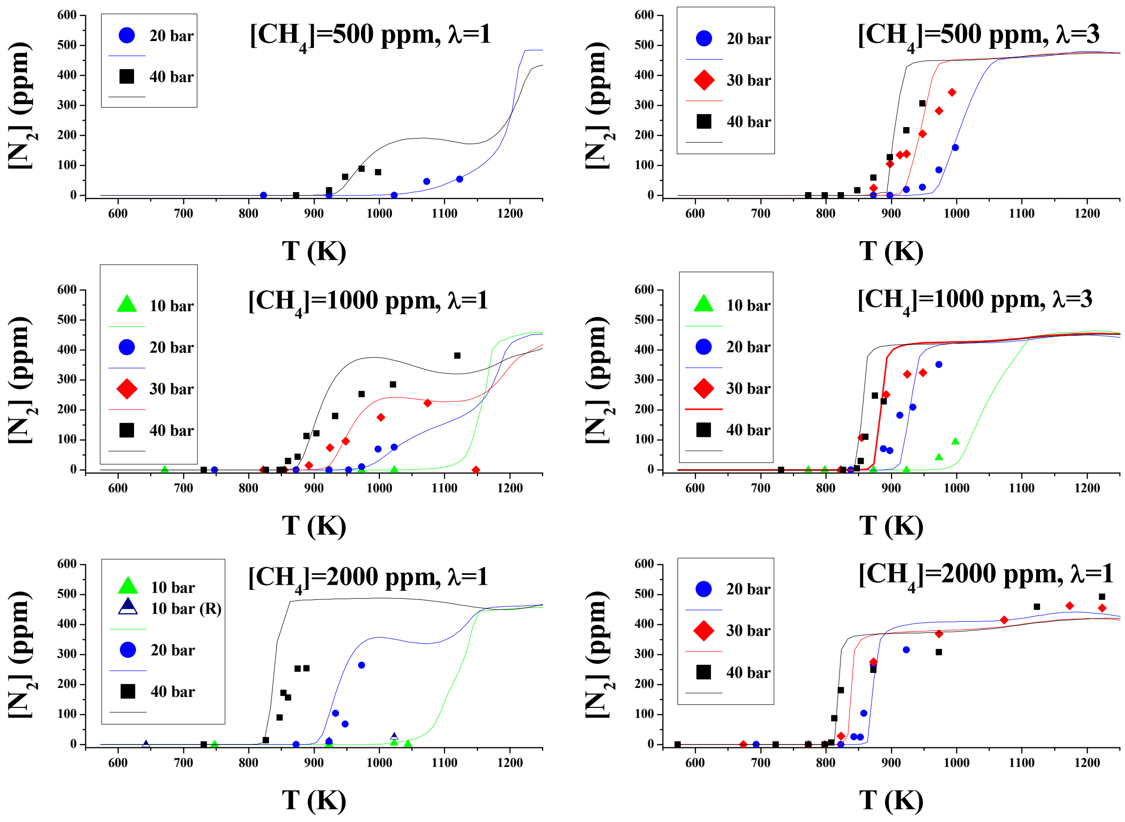

Figure S3. N_2 conversion as a function of temperature for different pressures (10 - 40 bar). Sets 2 - 6, 8 - 15 and 17 - 22 in Table 1. $[CH_4]$ = 500 ppm (a, b), 1000 ppm (c, d) and 2000 ppm (e, f). $[NH_3]$ = 1000 ppm. Left column λ = 1 and right column λ = 3. Ar as bath gas. Symbols are used to represent experimental data and lines for calculations. Residence time is defined by equation 2.

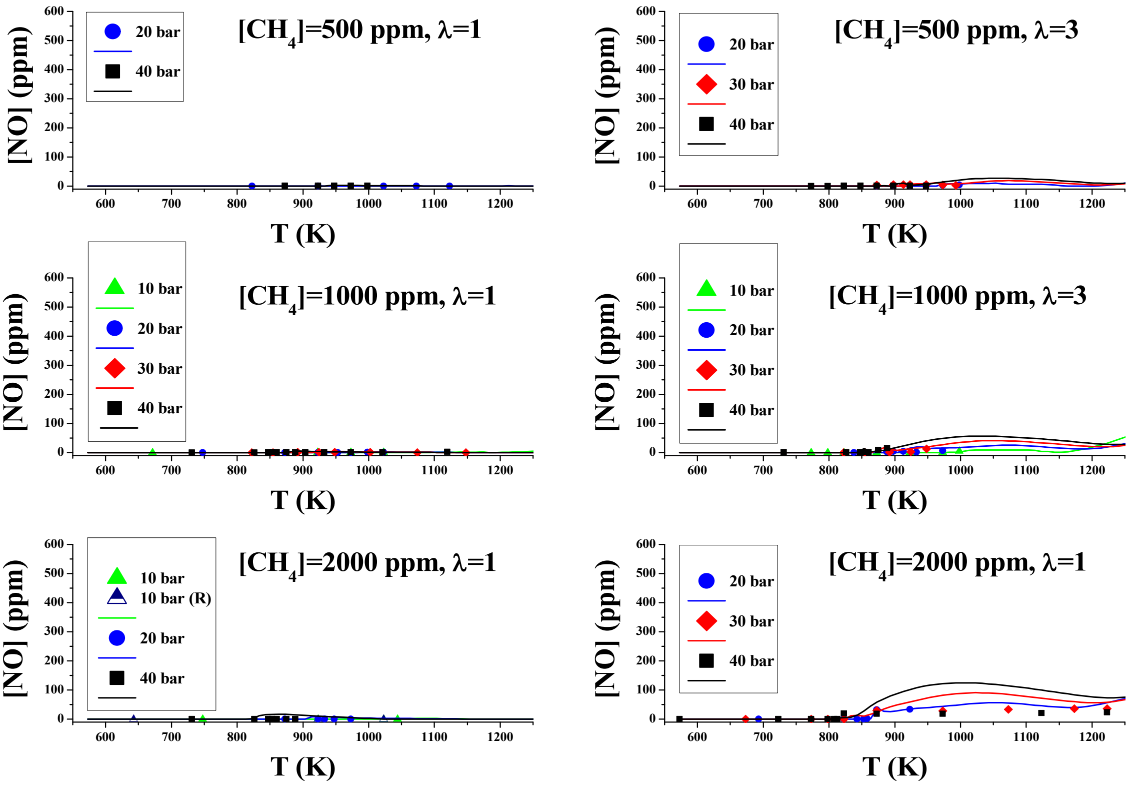

Figure S4. NO conversion as a function of temperature for different pressures (10 - 40 bar). Sets 2 - 6, 8 - 15 and 17 - 22 in Table 1. $[CH_4]$ = 500 ppm (a, b), 1000 ppm (c, d) and 2000 ppm (e, f). $[NH_3]$ = 1000 ppm. Left column λ = 1 and right column λ = 3. Ar as bath gas. Symbols are used to represent experimental data and lines for calculations. Residence time is defined by equation 2.

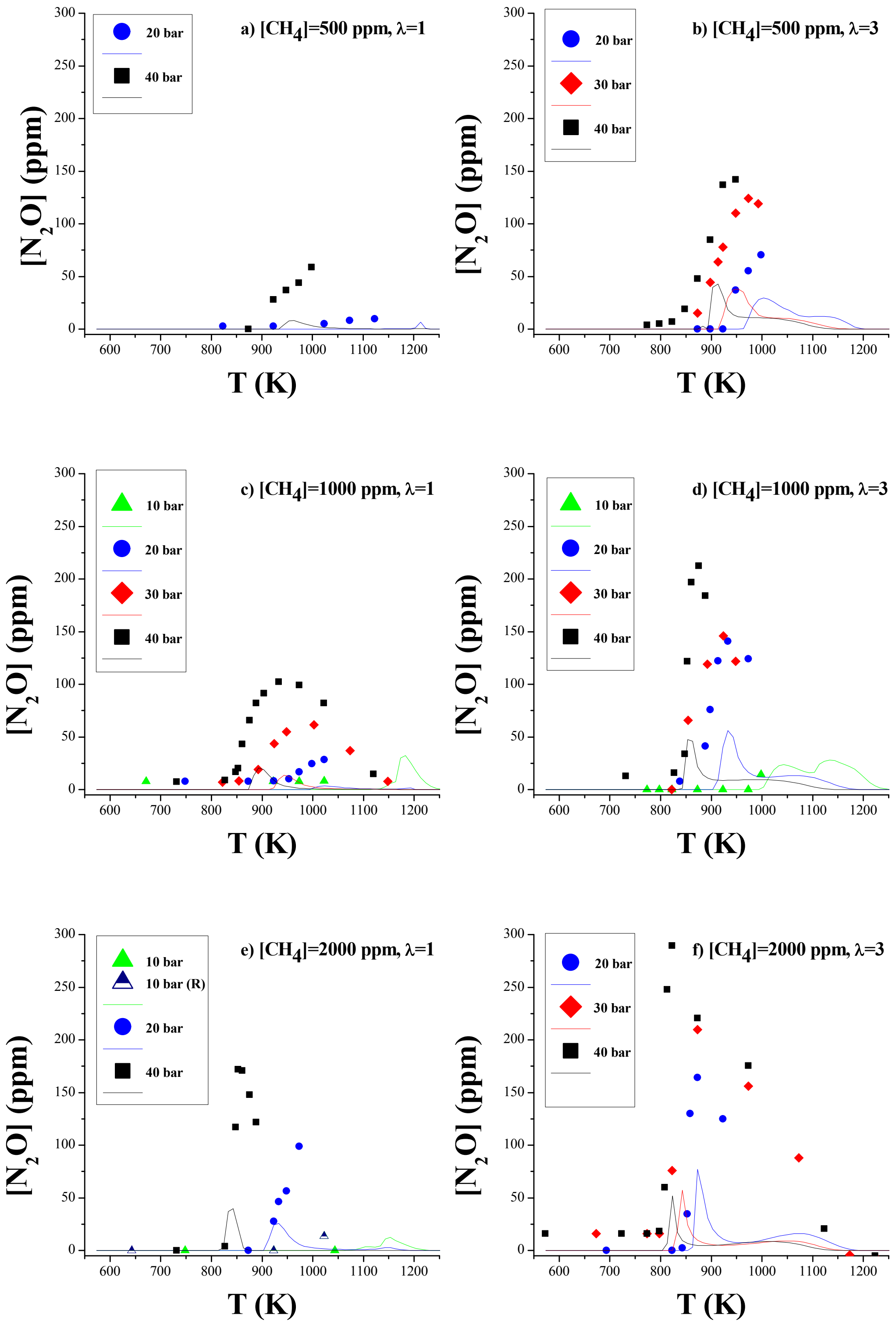

Figure S5. N_2O conversion as a function of temperature for different pressures (10 - 40 bar). Sets 2 - 6, 8 - 15 and 17 - 22 in Table 1. $[CH_4]$ = 500 ppm (a, b), 1000 ppm (c, d) and 2000 ppm (e, f). $[NH_3]$ = 1000 ppm. Left column λ = 1 and right column λ = 3. Ar as bath gas. Symbols are used to represent experimental data and lines for calculations. Residence time is defined by equation 2.

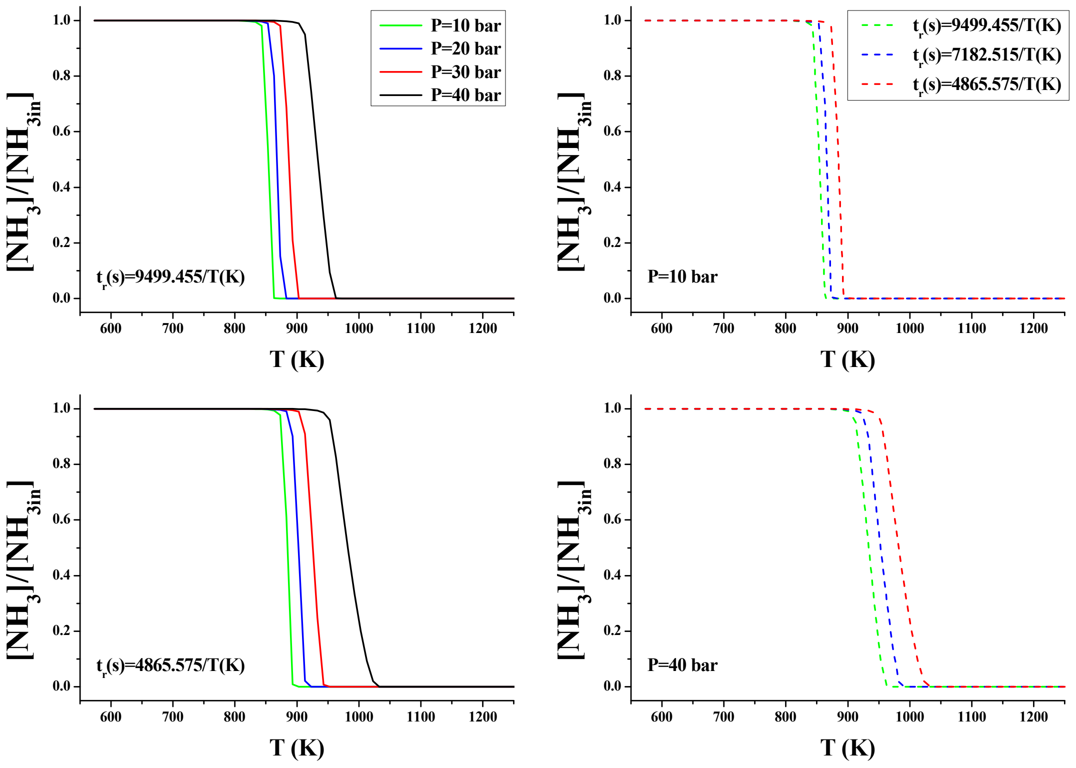

Figure S6. NH_3 evaluation through model calculations of the effect of pressure and gas residence time on the NH_3 (1000 ppm) conversion at $CH_4/NH_3 = 1$, under oxidizing conditions ($\lambda = 3$). Figure S6 (left) includes calculations made at different pressures (10, 20, 30 and 40 bar) with constant residence time. Figure S6 (right) includes calculations made at 10 and 40 bar and different residence times (9499/T, 7183/T and 4866/T (s)).

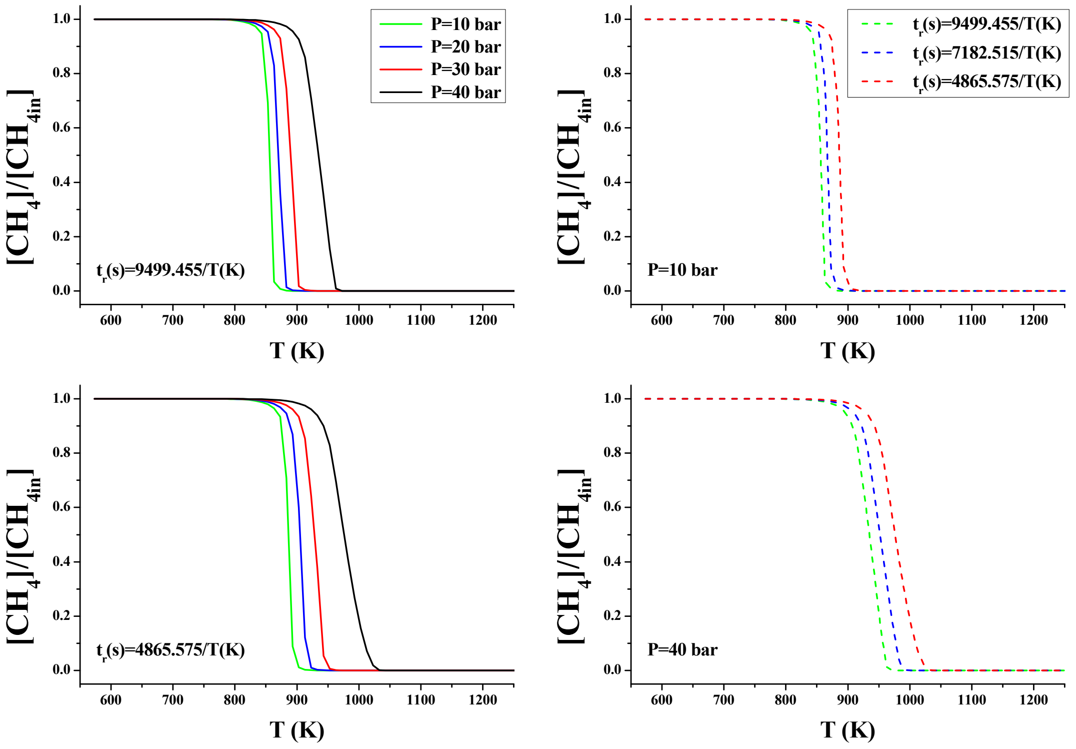

Figure S7. CH_4 evaluation through model calculations of the effect of pressure and gas residence time on the NH_3 (1000 ppm) conversion at CH_4/NH_3 = 1, under oxidizing conditions (λ = 3). Figure S7 (left) includes calculations made at different pressures (10, 20, 30 and 40 bar) with constant residence time. Figure S7 (right) includes calculations made at 10 and 40 bar and different residence times (9499/T, 7183/T and 4866/T (s)).
